# YOLO object detection models can locate and classify broad groups of flower-visiting arthropods in images

**DOI:** 10.1038/s41598-023-43482-3

**Published:** 2023-09-29

**Authors:** Thomas Stark, Valentin Ştefan, Michael Wurm, Robin Spanier, Hannes Taubenböck, Tiffany M. Knight

**Affiliations:** 1 German Remote Sensing Data Center (DFD), German Aerospace Center (DLR), Oberpfaffenhofen, Germany; 2 Department of Community Ecology, Helmholtz Centre for Environmental Research - UFZ, Halle (Saale), Germany; 3 German Centre for Integrative Biodiversity Research (iDiv) Halle-Jena-Leipzig, Leipzig, Germany; 4 Institute of Geography and Geology, University of Würzburg, Würzburg, Germany; 5 Institute of Biology, Martin Luther University Halle-Wittenberg, Halle (Saale), Germany

**Keywords:** Biodiversity, Population dynamics

## Abstract

Develoment of image recognition AI algorithms for flower-visiting arthropods has the potential to revolutionize the way we monitor pollinators. Ecologists need light-weight models that can be deployed in a field setting and can classify with high accuracy. We tested the performance of three deep learning light-weight models, YOLOv5nano, YOLOv5small, and YOLOv7tiny, at object recognition and classification in real time on eight groups of flower-visiting arthropods using open-source image data. These eight groups contained four orders of insects that are known to perform the majority of pollination services in Europe (Hymenoptera, Diptera, Coleoptera, Lepidoptera) as well as other arthropod groups that can be seen on flowers but are not typically considered pollinators (e.g., spiders-Araneae). All three models had high accuracy, ranging from 93 to 97%. Intersection over union (IoU) depended on the relative area of the bounding box, and the models performed best when a single arthropod comprised a large portion of the image and worst when multiple small arthropods were together in a single image. The model could accurately distinguish flies in the family Syrphidae from the Hymenoptera that they are known to mimic. These results reveal the capability of existing YOLO models to contribute to pollination monitoring.

## Introduction

Most crops and wild plant species rely on interactions with animal pollinators for reproduction^1^. Pollination is therefore a critical ecosystem services, which has an annual market value of more than $237 billion^2^. The threats to pollinators are not fully understood, which has led to urgent calls for more studies aimed at understanding how pollinators and plant-pollinator interactions change across environmental gradients (EU Pollinators Initiative, EC 2018).

Currently, most data on plant-pollinator interactions are acquired from field observations and collections of pollinators contacting reproductive parts of flowers. The identification of pollinators is a time-consuming step in this research. For example, it is typical for pollination ecologists to spend weeks in the field collecting observational data and arthropods, and many weeks or months afterwards pinning, sorting and identifying those arthropods using microscopy or DNA barcoding^2–4^. AI tools, in particular convolutional neural networks (CNNs) for image classification, have the potential to allow for efficient identification of plant-pollinator interactions from field images. Training these tools require a vast amount of annotated data, which can be met with the growing amount of digital data from citizen science platforms and media platforms that have been curated by taxonomic experts^5,6^.

Developments of applications for automated recognition have resulted in some outstanding products for plant identification, due to the consolidation of different machine learning methods^7,8^, the increase in image data availability^9^, and advances in computer hardware over the past decade. For example, the Flora Incognita app can currently distinguish more than 16,000 European plant species from images taken by citizens. Like plants, automated arthropod identification is challenged by the vast number of taxa to be classified, by the complexity of image backgrounds and variability in shooting angles, and by the fact that individuals of the same taxa vary in their morphology and individuals of different taxa sometimes look very similar^6,8^. Arthropod identification is also challenged by dealing with multiple individuals that overlap in the same image, different lighting conditions^6,10,11^, arthropods that blend in cluttered backgrounds, individuals that move very fast and/or are hidden by parts of plants^12^, and dramatic size differences between arthropod species.

Deep learning has revolutionized computer vision in many fields of natural sciences, such as medicine^13,14^, and remote sensing^15–17^. In the field of automatic identification of arthropods, convolutional neural networks (CNN) used for image classification are still in a pre-mature phase. However, it is acknowledged that they have the potential to revolutionize data collection^11,18^. The development of AI tools for arthropod classification started with a focus on particular taxa and/or the identification of museum specimens with a homogeneous image background. For example, pictures taken of arthropod wings in controlled settings (i.e., under the microscope) can identify several groups of bees^19^, butterflies^20,21^ and syrphid flies^22^. The application of CNNs on arthropod identification ranges from a few to multiple taxa and considers a growing number of images, for example, nine tiger beetles genera and 380 images^23^, nine groups of arthropods and nearly 2000 images^24^, ten butterfly species and nearly 18,000 images^21^, 36 bumble bee species and nearly 90,000 images^6^. Progress in this direction was carried out also in the citizen science and private sectors with the ongoing research in mobile apps like Seek, ObsIdentify, NABU Insektensommer and Google Lens.

Recent developments in camera hardware and image recognition have been applied to study insect pollination, typically focused on a single plant species or a single pollinator species. For example, Bjerge and colleagues^25^ use a custom camera system and CNNs to capture images and classify broad groups of arthropods that visit plants in the genus Sedum. Ratnayake and colleagues^12,26^ used Raspberry Pi hardware and CNNs to track the movement of individual honeybees, allowing for spatial monitoring and behavioral analysis of this important pollinator species in an agricultural setting. Several studies have introduced low-cost and open-source camera trap systems that are enabling efficient collection of insect images in field settings^27–29^. However, there is a need to develop tools that can classify flower-visiting arthropods that visit a wide variety of flowers in a broad geographic region, such as Europe.

**Figure 1:**
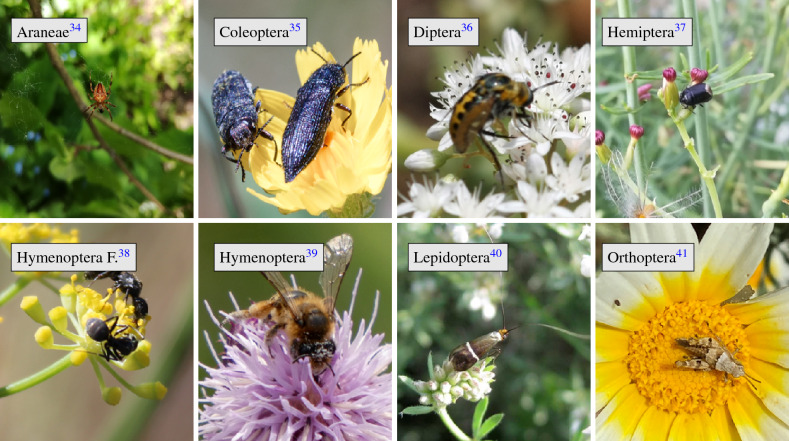
Example images for the eight groups in our dataset used for classification.

In Europe, four orders of arthropods perform the vast majority of pollination: Hymenoptera (bees and wasps), Diptera (flies), Lepidoptera (butterflies and moths), and Coleoptera (beetles)^30^. Further, there are four other taxa of arthropods that are more rarely seen on flowers and are not typically considered pollinators because they visit flowers so infrequently and/or deliver little pollen per visit^31^. These include the order Orthoptera (grasshoppers and leafhoppers), the order Hemiptera (true bugs), the order Araneae (spiders), and the family Formicidae (ants). Thus, it is necessary to consider the eight groups of arthropods to classify flower visitors as seen in Fig. 1.

In this study, we test whether a deep learning approach allows to localize and classify arthropods into these eight groups with sufficient accuracy. Therefore, we evaluated the performance of three light-weight object detection algorithms. The YOLO^32^ family of object detection algorithms offers multiple versions of its algorithm varying in a wide variety of use cases. In our experimental set-up, three different YOLO models were trained on more than 17,000 annotated images with the location of each arthropod being marked by bounding box coordinates. Using YOLO for object detection provides us with the benefit of detecting several arthropods in a single image, which is one of the reasons we want to explicitly test how well this task is performed. Additionally, we expected that one of the more difficult classifications will be of Dipterans in the family Syrphidae (hoverflies), which have mimicry of bees and wasps (Hymenoptera)^33^. Thus, we performed a separate test to see how well our model differentiates this family of flies from Hymenoptera on 1000 annotated images of hoverflies.

## Methods

### Dataset

The dataset covers the eight groups as seen in Fig. 1 and in Table 1: spiders (Araneae), beetles (Coleoptera), true flies (Diptera), true bugs (Hemiptera), bees and wasps (Hymenoptera), ants (Hymenoptera Formicidae), moths and butterflies (Lepidoptera), and crickets and grasshoppers (Orthoptera). Coleoptera, Diptera, Hymenoptera and Lepidoptera represent the most important pollinator groups of the dataset. These four orders make up the majority (aprox. 98%) of arthropods that visit flowers^30^. Ants (Hymenoptera in the family Formicidae) have been documented as pollinators in a few case studies, but have an insignificant role in pollination for most plants and can even be detrimental^42^. Likewise, Araneae, Hemiptera and Orthoptera might rarely be seen on flowers but will not play a significant role as pollinators, and can be detrimental as predators of pollinators^43,44^. Therefore, including these orders into the dataset prevents models from confusing them with the pollinator groups.

We used curated images indexed by the Global Biodiversity Information Facility (GBIF) database. A significant proportion of the images (96.77%) are sourced from the citizen science platforms, iNaturalist and Observation.org, which account for 53.86% and 42.91% of the contributions respectively. We extracted the occurrence data for our eight groups, and only kept the occurrence data that offered URLs with images. We then curated the datasets, focusing on species marked as present in Europe. For the orders Diptera, Coleoptera, Hymenoptera and Lepidoptera, we only sampled from known families of flower visitors. There was an uneven distribution of images across families within an order and across species within a family. For example, within the order Diptera, the Syrphidae family has many more images indexed by GBIF than other Dipteran families. Within the Syrphidae family, the common and widespread species Eristalis tenax is overrepresented compared to other species. Our methods therefore aimed to capture the taxonomic (and likely morphological) diversity of images that capture the representative range for each order, and to use random sampling in order to avoid selection biases. Specifically, for families consisting of more than 200 species, we randomly selected 200 species, and randomly choose one image per species. For families with fewer than 200 species, we randomly selected multiple images per species.

There is a high variability in the dataset with images of arthropods from different angles, exposure, focus, framing sizes and focal length. Also, there are various image backgrounds from artificial to natural ones. These characteristics are representative of those found in the iNaturalist dataset^45^. Images are captured using a variety of devices, from professional cameras to mobile phones. The citizen science platforms encourage users to crop their raw images such that arthropods appear more prominent. However, the degree to which this is applied varies among users, resulting in images where the arthropods occupy a significant portion of the image area in some cases, and a smaller portion in others. This introduces an inherent variability in the framing or ‘crop level’ of the insects across the dataset. The average area occupied by the insects, expressed as a proportion of the total image area (relative bounding box area), is 0.34 (Table 1), with an interquartile range between 0.10 and 0.54. Thus, our methods should allow for results that can be effectively extrapolated to further images captured by citizen scientists.

Each arthropod in these images was manually annotated with a bounding box by entomologists using the open source VIA Annotation program^46^. We discarded images that were identified incorrectly, showed larva and pupa stages, the arthropod was missing, and/or did not show the entire body of the arthropod. Our initial dataset consisted of 20,505 images of which we discarded 2,797 images. Table 1 shows the final sample sizes for training, validation, and testing. We split our dataset into a group-balanced validation and testing dataset. We used 210 images per group for testing and validation, and remaining images for training. The testing dataset was never shown to the object detection architecture, while the validation dataset was used for monitoring the model performance during the training procedure and for determining when to stop training in order to avoid overfitting.

### YOLO

We assess the potential of three one-stage object detection models. We chose light-weight models with the intention that future applications will require the algorithms to run on mobile devices. Simultaneously, a trade-off must be made in which the detection model must be capable of performing successful classification tasks. YOLO^32^ is a one stage object detector, since it can integrate object classification and object location into one network architecture. The YOLO object detection algorithm divides the input image into SxS grids and each grid cell is responsible for predicting the object centered in that grids cell. Each grid cell predicts a number of bounding boxes and their corresponding confidence scores. Formally, confidence scores for each prediction are defined. This indicates the likelihood of objects and shows confidences of its prediction. At the same time, regardless of the number of boxes, conditional probabilities are predicted in each grid cell. It should be noted that only the contribution from the grid cell containing an object is calculated. While the original YOLO version was capable of real time detection, one of its biggest drawbacks was its performance on small objects. YOLOv2^47^ introduced batch normalization, anchor points and the use of higher resolution images. YOLOv3^48^ built upon previous models by adding an object score to bounding box prediction, added connections to the backbone network layers and made predictions at three separate levels of granularity to improve performance on smaller objects. YOLOv4^49^ introduced further improvements in its backbone, feature aggregation, and its head for the detection step. Furthermore a set of data augmentation and activation functions could improve results.

### YOLOv5

YOLOv5^50^ was originally developed in PyTorch and not DarkNet like its predecessors. YOLOv5 offers multiple models, the difference between them is the trade-off between the size of the model and inference time. There are six different architectures specific to YOLOv5 (nano, small, medium, large and extra-large), they are distinguished from each other by the depth of the network, primarily the backbone network with the number of convolutional layers. In YOLOv5 the cross stage partial (CSP) networks are used as a backbone to extract features. In YOLOv5 PANet^51^ is used for the neck to get feature pyramids. The model head is mainly used to perform the final detection part. YOLOv5 applies anchor boxes on features and generates the final output vectors with probabilities, an objectness score, and bounding boxes. YOLOv5 authors decided to use a combination of Leaky ReLU and Sigmoid activation functions. In YOLOv5, the default optimization function for training is stochastic gradient descent (SGD) and the default loss function used is the binary cross entropy with logits.

### YOLOv7

In YOLOv7^52^ multiple incremental improvements were made from its previous versions. Extended efficient layer aggregation (E-ELAN) is used as a computational block, which enables quicker back-propagation and speeds up training and inference time. YOLOv7 scales the network depth and width while concatenating layers together. Reparameterization techniques, which involve averaging a set of model weights, creates models that are more robust in learning patterns. The YOLOv7 authors use gradient flow propagation paths to see which modules in the network should use reparameterization strategies and which should not. For the final predictions the authors use an auxiliary head with different levels of supervision, settling on a coarse to fine definition where supervision is passed back from the lead head at different granularities.

### Training set-up

**Figure 2:**
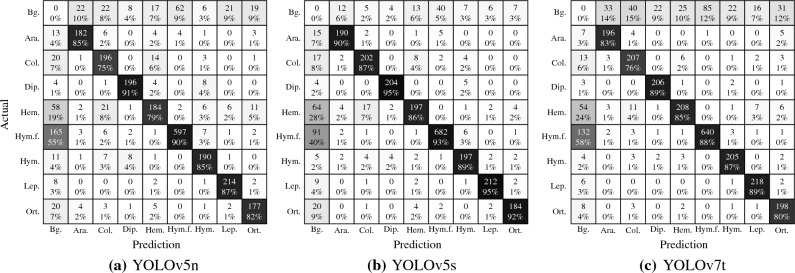
Confusion matrix for all three models (**a**) YOLOv5n, (**b**) YOLOv5s, and (**c**) YOLOv7t. Columns show the predicted values and rows the actual label values.

**Figure 3:**
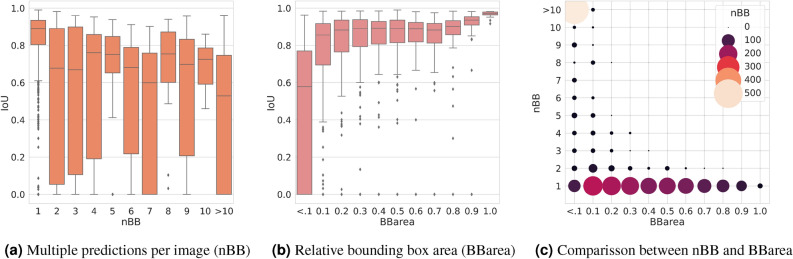
The figures show results for YOLOv7t, (**a**) IoU depending on the number of bounding boxes per images [nBB], (**b**) IoU depending on the relative area of the bounding box to the image size [BBarea], and (**c**) counts of the number of bounding boxes in our dataset for each combined category of nBB and BBarea. The size and color of each dot represents the number of bounding boxes.

**Figure 4:**
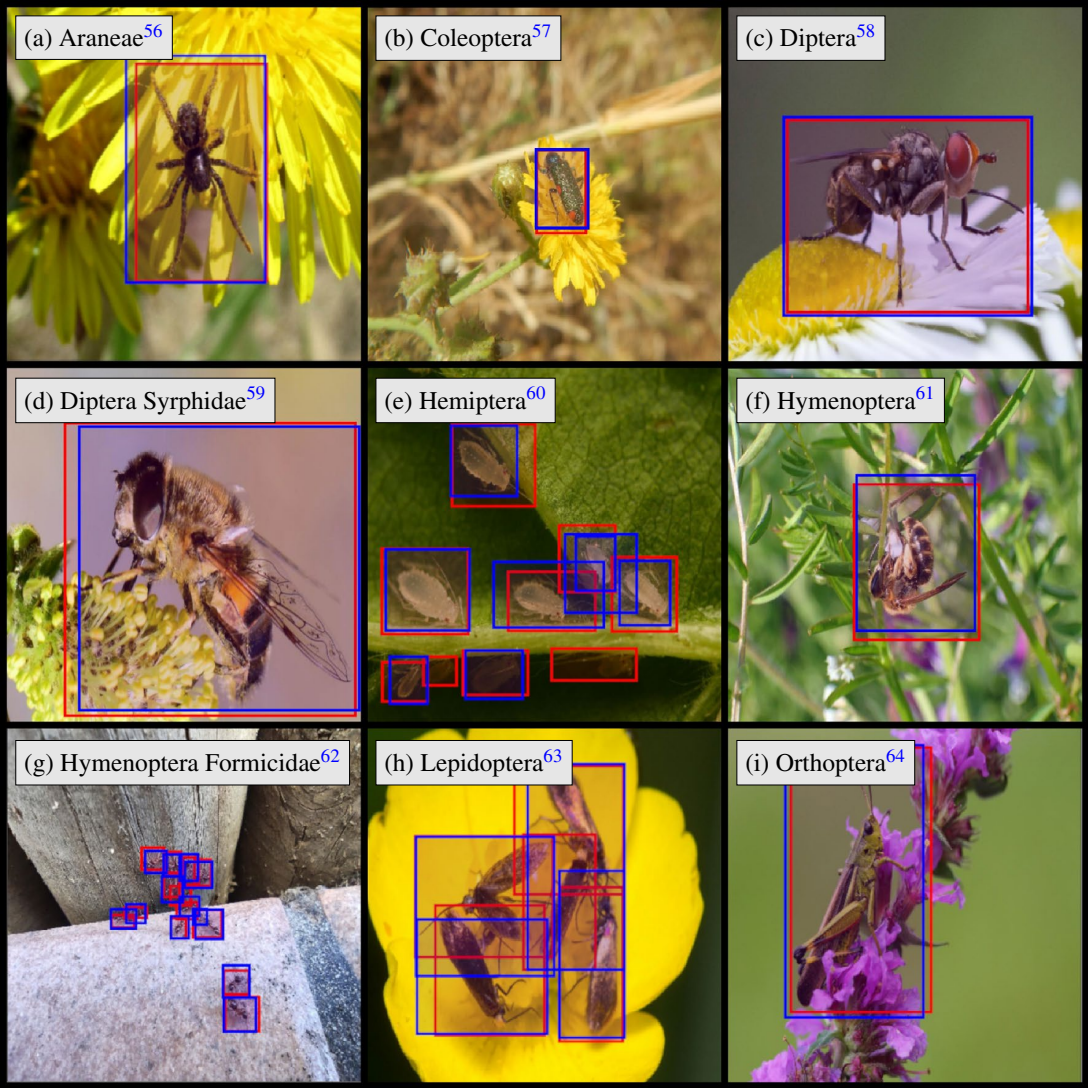
Predictions (blue) and labels (red) for nine images of arthropods in our dataset using the YOLOv7t model.

We selected three models YOLOv5n (nano), YOLOv5s (small), and YOLOv7t (tiny) to compare to each other. All three models offer less than ten million trainable parameters and are capable for real time detection even on CPUs and low end hardware GPUs. The models were trained using an input image size of 640*640 pixels in order to use a batch size of eight for all models, which was the highest value allowed by the available hardware. We decided not to train the models from scratch, but to use pre-trained weights from the COCO^53^ dataset which are provided with each YOLO repository, since this can result in more robustness in the model performance^54^. All models are trained using three epochs to “warmup” the optimizer, since this can help with faster convergence^55^ and with the same set of hyper-parameters for 300 epochs (See supplementary information Table 1), while the best model is determined by its fitness using a weighted average of the mean average precision at various intersection over union thresholds. To evaluate our models we used the following metrics: overall accuracy, precision, recall, intersection over union (IoU), and the false positive rate. The confusion matrix is formed to calculate group specific metrics. All three models provide a prediction confidence level and an IoU score threshold. Using the findings for the overall accuracy, the best possible combination was identified using a grid search from 10% to 90% in 10% increments for both thresholds (See Figs. 1–4 supplementary information). We report the accuracies depending on the highest overall accuracies achieved by the threshold grid search. The computations for this work were done using resources from the Leipzig University Computing Center which granted us quota access to compute nodes with eight Nvidia RTX2080Ti 11 GB each.

**Table 1:** The sample sizes in the dataset used for training and testing the object detection algorithm. While the training dataset shows some imbalance in its image count of the groups, the validation and testing dataset are sampled evenly for each group.

Group	Images	Bounding boxes	Relative bounding box size	Validation images	Test images	Training images
Araneae	1523	1578	0.38	210	210	1103
Coleoptera	2336	2560	0.23	210	210	1916
Diptera	2401	2468	0.49	210	210	1981
Hemiptera	1711	2247	0.26	210	210	1291
Hymenoptera F.	1051	3368	0.08	210	210	631
Hymenoptera	2461	2543	0.39	210	210	2041
Lepidoptera	4577	4749	0.36	210	210	4156
Orthoptera	1649	1705	0.48	210	210	1229
	17,709	21,218	0.34	1680	1680	14,348

## Results

We discovered that, in general, all three models (YOLOv5n, YOLOv5s, and YOLOv7t) were able to distinguish the order of flower visitors in images with high accuracies of up to 96 percent. As a result, we show that this method of categorization is capable of both finding the location of an arthropod within a picture and identifying it to its group.

For YOLOv5n the highest overall accuracy acheived 94.50%; this was achieved with a combination of a 20% confidence and 50% IoU threshold. YOLOv5s achieved 96.24% accuracy with a combination of a 30% confidence and 60% IoU threshold. YOLOv7t achieved 95.08% accuracy with a combination of 10% confidence and 30% IoU threshold. Despite their modest model sizes of less than 10 million trainable parameters, all models, YOLOv5n, YOLOv5s, and YOLOv7t, were able to recognize the order of flower visitors in images. YOLOv5s is approximately 3.8 times larger than YOLOv5n and achieves a 1.7% greater overall accuracy. YOLOv5s and YOLOv7t are nearly the same size and have an overall accuracy difference of less than 1.2%. The remaining accuracy metrics are shown in Table 2. Precision indicates how accurate predictions are, whereas recall measures the proportion of ground truth instances that were correctly identified by the model. The false positive rate (FPR) is the proportion of negative cases in data that were incorrectly recorded as positive. Note that YOLOv5n and YOLOv7t get comparable results, with the exception of the higher recall score for YOLOv7t. The IoU is determined for all true positives by taking the mean of all predicted bounding boxes in an image. All YOLO versions scored a mean IoU of above 78%.

**Table 2:** Overview on all metrics of the three models YOLOv5n, YOLOv5s, and YOLOv7t.

	Number parameters (m)	CPU inference [sec/img]	GPU inference [sec/img]	Conf. & IoU Threshold	Overall accuracy	Precision	Recall	False positive rate	IoU
YOLOv5n	1.9	0.1893	0.0552	0.2 & 0.5	0.9450	0.8427	0.8305	0.0309	0.7831
YOLOv5s	7.2	0.4833	0.0554	0.3 & 0.6	0.9624	0.9088	0.8692	0.0209	0.8052
YOLOv7t	6.2	0.4047	0.0734	0.1 & 0.3	0.9508	0.8449	0.8907	0.0283	0.8026

The confusion matrix of the predictions on the test dataset for each of the three models is shown in Fig. 2. The matrix shows the eight groups in our dataset, in addition a background group (Bg.) is added to count false positives and false negatives as well. We show the confusion matrix using the confidence and Iou threshold from Table 2 scoring the highest overall accuracy. The confusion matrix visualizes and summarizes the classification of the algorithm’s performance, with each row of the matrix representing examples in an actual group and each column representing occurrences in a predicted group. Since there may be many label bounding boxes for a given picture, the total number of images in a row may exceed 210 even if each row contains exactly 210 images from the test dataset in Table 1. All three YOLO models in Fig. 2, YOLOv5n (Fig. 2a), YOLOv5s (Fig. 2b), and YOLOv7t (Fig. 2c), exhibit similar behavior, with the diagonal indicating true positive scores always greater than 75%. It is worth noting that all three models had some missed detections for the Hemiptera and Hymenoptera Formicidae groups. As seen in Table 1 this can be linked to small bounding boxes and several overlapping labels per picture. Both groups share noticeable smaller bounding boxes as well as a higher ratio of bounding boxes per image compared to other groups. As shown in the first row of the confusion matrix in Fig. 2c, YOLOv7t suffers from more false positives than its YOLOv5 counterparts.

Aside from comparing metrics between YOLO versions, we also look closely at outcomes for detecting several objects in a single image and images with small bounding box labels as seen in Fig. 3. The ability to recognize many arthropods of various types in a single image is a significant advantage of employing object detection rather than scene classification approaches. In these challenging situations, we particularly examine the IoU metric as the accuracy of the bounding box placement as a very relevant indicator of the models performance. The results on images grouped by their number of labels per image is shown in Fig. 3a, and the results for predictions depending on the relative area of its bounding box in Fig. 3b. In Fig. 3a the IoU score is shown for images with the number of detections ranging from one to ten, while the last group consists of all images with more than ten detections. In Fig. 3b the IoU score is shown depending on the relative area of the bounding box in relations to its image size, the higher the value of BBarea the larger is the bounding box. The IoU measurements demonstrate a significant drop in their values when the number of labels per image increase. The same effect can be observed when dealing with small bounding boxes. In Fig. 3c we compare the number of bounding boxes per images and the relative area of the bounding boxes is shown. We can see that a larger number of bounding boxes on images also corresponds to a lower relative bounding box size, while larger bounding box areas are typically found on images where there is only one single bounding box. It is also worth noting that in Fig. 3b,c the group Hymenoptera Formicidae are responsible for the bulk of the smaller bounding boxes, and as a result, they have a considerable influence on the distribution of bounding boxes. This is especially noticeable in Fig. 3c, where the large circle in the upper left corner highlights the impact.

We carried out an additional test in which we compared the detection results of the family Syrphidae within Diptera using the YOLOv7t model. Table 3 shows accuracy metrics for two combinations of confidence and IoU thresholds, where the first, using 10% confidence & 30% IoU was used due to scoring the highest overall accuracy from Table 2 and the second combination of 30% confidence & 60% IoU, which scores the highest overall accuracy for the Syrphidae dataset (See supplementary information Fig. 5). While both experiments demonstrated exceptional results, it can be argued that using 30% confidence & 60% IoU was slightly superior, as it exhibited a higher level of overall accuracy, precision, and false positive rate, however, both experiments should be deemed highly successful as they both correctly classified Syrphidae. Table 4 shows the confusion matrix for threshold combination of 30% confidence & 60% IoU. The confusion matrix is computed using the 1061 images of Syrphidae, to test whether the YOLOv7t model misclassifies the family into different groups, specifically the Hymenoptera order that they mimic. From Table 4 we can see that of the 1061 Syrphidae, only 55 are classified as Hymenoptera, which is the highest amount of the misclassified detections, as well as 39 labels of bounding box which were not recognized at all. Successful classification examples can be seen in Fig. 4d,f.

**Table 3:** Accuracy metrics using YOLPv7t for classifying images of arthropods in the family syrphidae into their appropriate group Diptera using two threshold combinations.

	Conf. &	
	0.1 & 0.3	0.3 & 0.6
OA	0.9655	0.9729
Precision	0.9502	0.9886
Recall	0.9171	0.8954
FPR	0.0748	0.0707
IoU	0.8312	0.8311

**Table 4:**
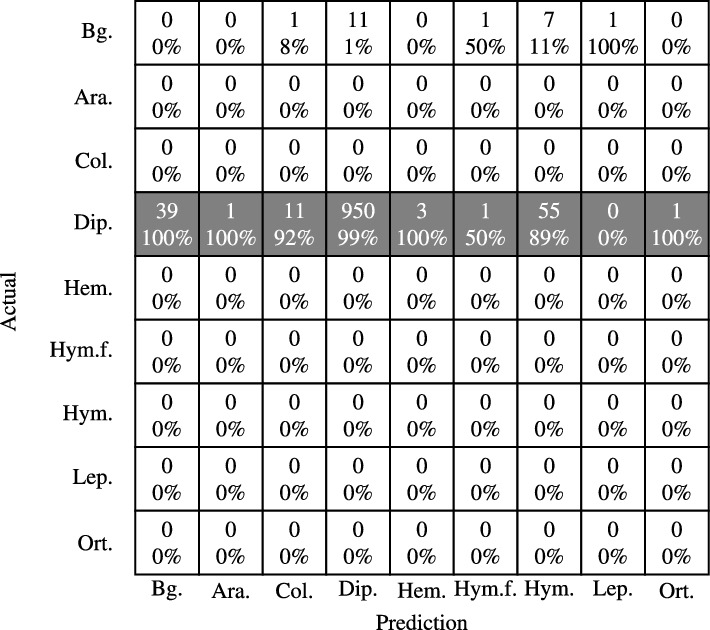
Confusion matrix using YOLOv7t for the family Syrphidae within Diptera for a combination of 10% confidence and 30% IoU threshold.

In Fig. 4 some of the predictions made by the YOLOv7t model are depicted, including predictions of images with multiple arthropods. The figure shows nine images of seven orders from our dataset. While the YOLOv7t model correctly classifies all arthropods to its specific group, in cases with a large count of small labels there are some false negatives as seen in Fig. 4e,g,h. Figure 4c,d,f shows that the YOLOv7t correctly differentiates between Diptera-Syrphidae and Hymenoptera.

## Discussion

Identifying flower-visiting arthropods is a challenging task, especially when one considers the trade-off that must be made between the amount of processing power necessary and the degree of accuracy that must be achieved in order to get acceptable results. The YOLO versions used in this study, v5 and v7, provide several options for models that would be suited to this problem. We tested three light-weight models that could be deployed in a field setting for automated pollinator monitoring: YOLOv5 with the smallest sizes, “nano” (n) and “small” (s), and YOLOv7 with the smallest size, “tiny” (t). We have found that the results of YOLOv5s and YOLOv7t are quite similar. The parameter counts of YOLOv5s and YOLOv7t are very comparable. YOLOv5n, on the other hand, has a much reduced size and this likely explains its lower scores as we found that the number of parameters influenced all of the accuracy metrics.

The advantage of using object detection rather than scene classification is not only in determining the location of the arthropod on the photos, but also in recognizing several arthropods in one image. This is important for the development of future automated pipelines for pollinator monitoring because it allows all individuals visiting a flower to be counted and identified. However, we observed that the model performance drops slightly when there are multiple arthropods in an image (Fig. 3). This is because models are faced with the challenge of small objects, since images with multiple arthropods typically contain smaller bounding boxes than images of a single arthropod. When there is only one arthropod to locate in the image, both YOLOv5s and YOLOv7t give similar results.

The high accuracy of the model in distinguishing the Syrphidae from Hymenoptera demonstrates the potential of the model to be a reliable tool. It is a relatively easy job to distinguish between some of the morphologically distinct groups in our study, such as Lepidoptera and Coleoptera. However, distinguishing Dipterans in the family Syrphidae from Hymenopterans is a challenging task, as Syrphidae mimic the morphological appearance of bees and wasps in the order Hymenoptera^65^. The high performance of our model in this task suggests great promise for future development of models, which will aim to classify each image to the lowest taxonomic level that an expert could with high accuracy.

A major challenge in understanding trends and drivers in pollinators and pollination is insufficient monitoring, as our current data is geographically biased and uses inconsistent methodology. Mobilizing citizen scientists is one solution that works well for charismatic taxa that are easy to identify, such as butterflies. Indeed, more than 3000 transects are monitored for butterflies each year by citizens throughout Europe^66,67^. However, expanding such monitoring to other pollinating taxa is difficult, as most citizens are not interested in learning to distinguish amongst Dipteran families or in killing the arthropods they observe so that they can be identified by experts. The results presented here are working towards a future in which pollinators and plant-pollination interactions are consistently and non-destructively monitored across broad spatio-temporal scales using time-lapse cameras set to photograph flowers in the field and then using CNNs to classify arthropods in those images. In Europe, such monitoring schemes could be possible in existing distributed networks such as the European Long Term Ecosystem Research (eLTER) network. However, it is an open question how well our CNN models developed in this manuscript, which use GBIF-indexed images, will perform on classifying images that are collected by a time-lapse camera. Testing how well our models perform on such an out-of-sample dataset is an important direction for future research.

Object detection algorithms using deep learning have proven to be effective in achieving high accuracy in a variety of tasks, making them a popular choice for machine learning practitioners. However, the complexity and computational requirements of incredible deep networks can make it challenging to deploy them in real-world applications where resources may be limited or the need for quick decision-making is crucial. Given this, it’s important to consider deep learning models using fewer trainable parameters that may not have the same level of accuracy but are more practical for deployment in the real world. While deeper networks still hold potential for improving accuracy, it may be necessary to balance that with considerations of practicality and feasibility in real-world settings.

Our study represents a significant advancement in the field of AI-assisted identification of European flower visitors, as we encompass a wide range of arthropod taxa in our training data set that occur on a wide variety of floral backgrounds (and other complex backgrounds). However, we see this as an initial step in the progress that is needed for automated insect identification, as many ecological questions and monitoring aims will require identification at lower taxonomic levels. Entomologists that specialize in taxonomy would hypothesize that species-level identification might not be possible, as identifying features at the species level are not visible on field images, such as features of the genitalia, placement of hairs on the legs, etc. However, traditional convolutional neural networks (CNNs) have the potential to surpass human-level classification in detecting pollinators by identifying intricate features and patterns that may elude the human eye^68^. Thus, we see testing the potential and limits of CNNs for family, genera, and species level identification of insect pollinators as an exciting next step in this line of research.

## Conclusion

In the near future, object detection will be a vital component of pollinator monitoring, allowing ecologists to have near real time data on pollination across broad spatial gradients. To correctly evaluate changes in plant-pollinator interactions across space and time, it will be essential that we continue to develop this technology, as well as data sets that can be utilized for training models. We have shown that the YOLO object detection models can be used to classify arthropod orders and localize the arthropods with bounding boxes. Localization is an important task to consider for the development of AI applications in pollination ecology because quantifying plant-pollinator networks requires also abundance information (counts of individual arthropods). We chose to evaluate three different light YOLO models: YOLOv5-nano, YOLOv5-small, and YOLOv7-tiny, which all have less than ten million trainable parameters. This is important for the future development of edge AI applications in pollination ecology because lighter models can be deployed to run detection in real time on edge devices under field conditions, with limited computational power and limited energy supply. The YOLOv5-nano model was selected for use as the foundation because it provides the lowest number of parameters while still delivering a satisfactory compromise between precision and speed. YOLOv5-small and YOLOv7-tiny models share similar number of parameters and, therefore do not differ much in their results. The results scale almost linearly with the number of parameters. Therefore, a suitable model type can be selected from our tests depending on the capabilities of future low-cost hardware setup to monitor pollination.

### Supplementary Information

Supplementary Information.

## Data Availability

The datasets generated during and/or analyzed during the current study are available from the corresponding author on reasonable request. The scripts to run all experiments are publicly available through our GitHub page stark-t/PAI.
