# Supplementary material for: YOLO object detection models can locate and classify broad groups of flower-visiting arthropods in images
Source: Sci Rep. 2023 Sep 29;13:16364. doi: 10.1038/s41598-023-43482-3 (PMC10541899; doi:10.1038/s41598-023-43482-3)
Supplement: Supplementary file 1 — Supplementary Information. [file 41598_2023_43482_MOESM1_ESM.pdf]

YOLO object detection models can locate and classify broad groups of flower-visiting arthropods in images

Thomas Stark[1,*], Valentin Ștefan[2,3], Michael Wurm[1], Robin Spanier[1], Hannes Taubenböck[1,4], and Tiffany M. Knight[2,3,5]

[1]German Aerospace Center (DLR), German Remote Sensing Data Center (DFD), Oberpfaffenhofen, Germany.
[2]Department of Community Ecology, Helmholtz Centre for Environmental Research - UFZ, Halle (Saale), Germany.
[3]German Centre for Integrative Biodiversity Research (iDiv) Halle-Jena-Leipzig, Leipzig, Germany.
[4]Institute of Geography and Geology, University of Würzburg, Würzburg, Germany
[5]Institute of Biology, Martin Luther University Halle-Wittenberg, Halle (Saale), Germany
[*]thomas.stark@dlr.de

ABSTRACT

Develoment of image recognition AI algorithms for flower-visiting arthropods has the potential to revolutionize the way we monitor pollinators. Ecologists need light-weight models that can be deployed in a field setting and can classify with high accuracy. We tested the performance of three deep learning light-weight models, YOLOv5nano, YOLOv5small, and YOLOv7tiny, at object recognition and classification in real time on eight groups of flower-visiting arthropods using open-source image data. These eight groups contained four orders of insects that are known to perform the majority of pollination services in Europe (Hymenoptera, Diptera, Coleoptera, Lepidoptera) as well as other arthropod groups that can be seen on flowers but are not typically considered pollinators (e.g., spiders-Araneae). All three models had high accuracy, ranging from 93% to 97%. Intersection over union (IoU) depended on the relative area of the bounding box, and the models performed best when a single arthropod comprised a large portion of the image and worst when multiple small arthropods were together in a single image. The model could accurately distinguish flies in the family Syrphidae from the Hymenoptera that they are known to mimic. These results reveal the capability of existing YOLO models to contribute to pollination monitoring.

Supplementary Materials

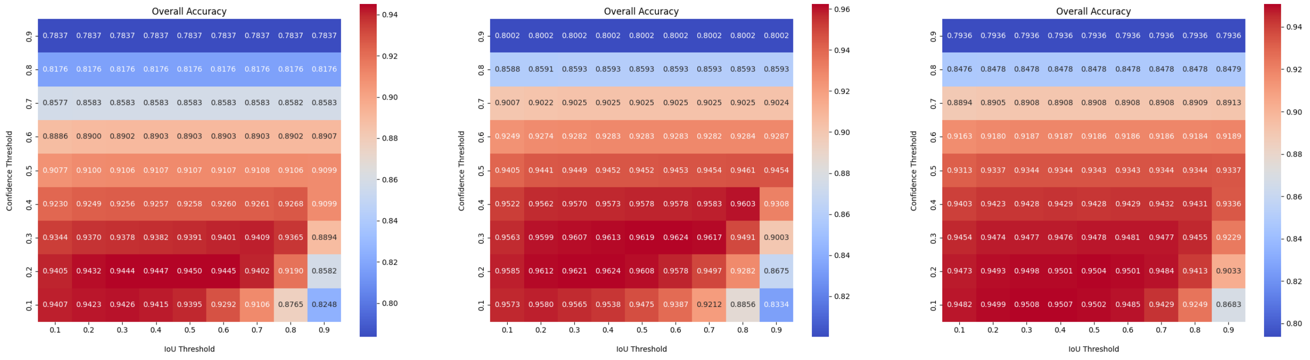

(a) OA YOLOv5n **(b)** OA YOLOv5s **(c)** OA YOLOv7t

Figure 1. Overall accuracy for 81 threshold combinations of the confidence and IoU variable. The overall accuracy for YOLOv5n is highest for 20% confidence and 50% IoU, for YOLOv5s highest for 30% confidence and 60% IoU, and for YOLOv7t highest for 10% confidence and 30% IoU is highest if the confidence threshold is 90% for all three models.

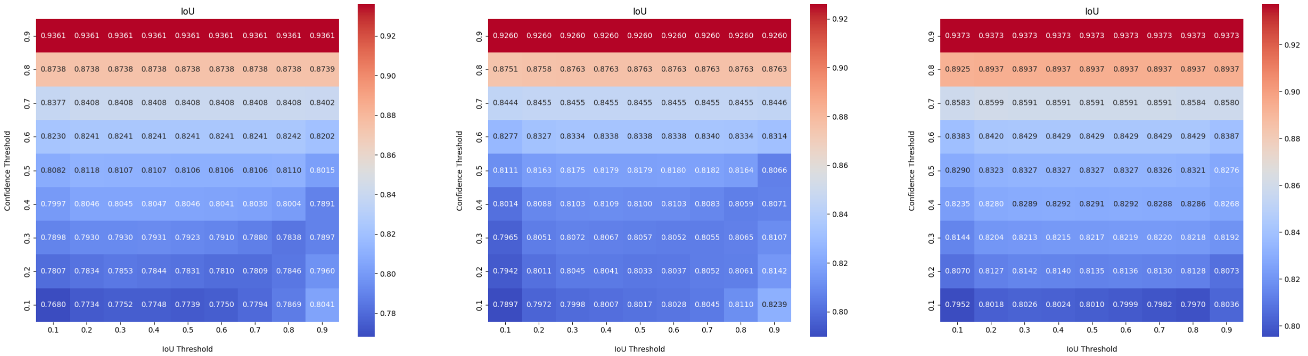

(a) IoU YOLOv5n **(b)** IoU YOLOv5s **(c)** IoU YOLOv7t

Figure 2. IoU metric for 81 threshold combinations of the confidence and IoU variable. IoU score is highest if the confidence threshold is 90% for all three models.

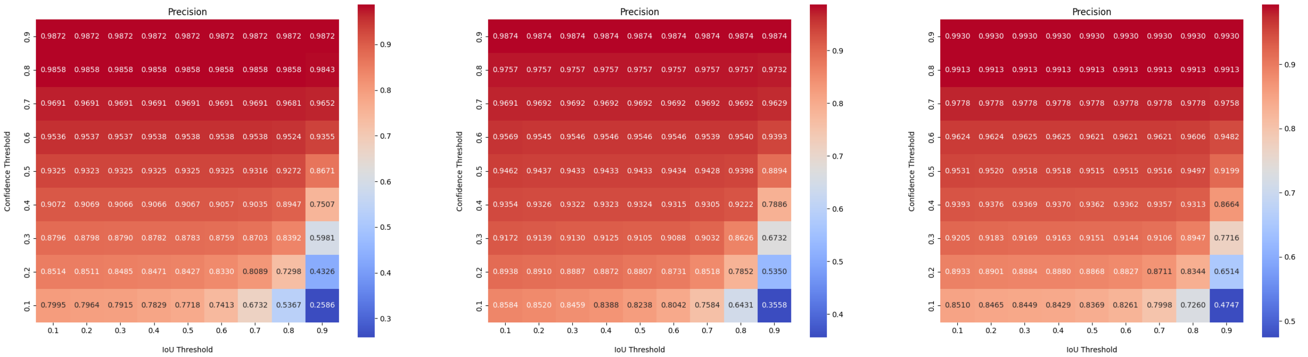

(a) Precision YOLOv5n **(b)** Precision YOLOv5s **(c)** Precision YOLOv7t

Figure 3. Precision metric for 81 threshold combinations of the confidence and IoU variable. The precision score is highest if the confidence threshold is 90% for all three models.

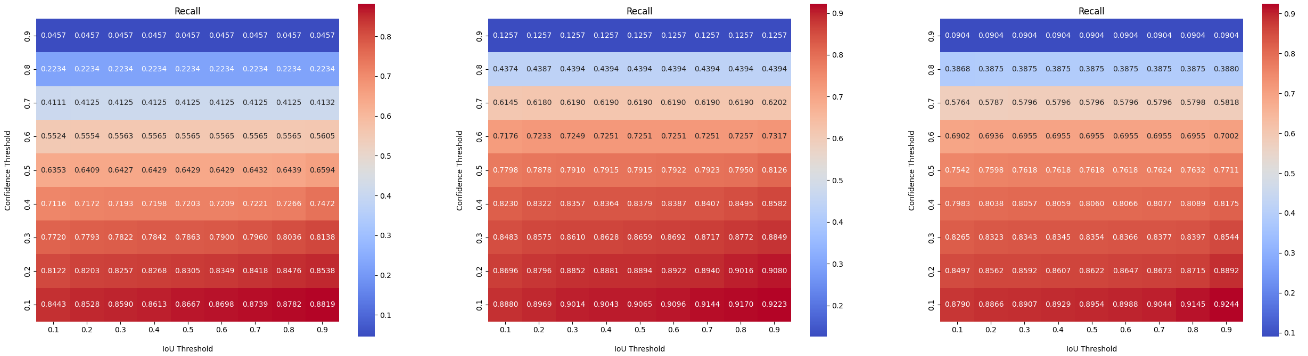

(a) Recall YOLOv5n **(b)** Recall YOLOv5s **(c)** Recall YOLOv7t

Figure 4. Recall metric for 81 threshold combinations of the confidence and IoU variable. The recall score is highest if the confidence threshold is 10% and 90% IoU.

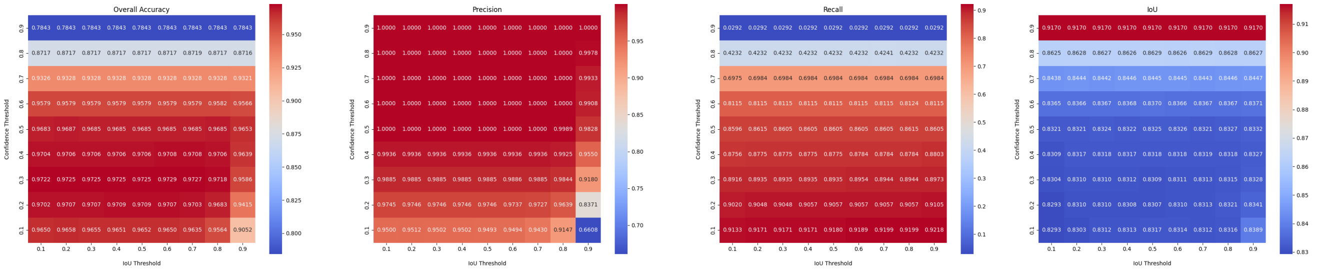

(a) OA syrphidae **(b)** Precision syrphidae **(c)** Recall syrphidae **(d)** IoU syrphidae

Figure 5. Overall accuracy, precision, recall, and IoU score for the grid search for the Diptera-Syrphidea family dataset.

Table 1. YOLO hyperparameters.

Parameter	Value	Description
lr0	0.01	initial learning rate (SGD=1E-2, Adam=1E-3)
lrf	0.1	final OneCycleLR learning rate (lr0 * lrf)
momentum	0.937	SGD momentum/Adam beta1
weight_decay	0.0005	optimizer weight decay 5e-4
warmup_epochs	3.0	warmup epochs (fractions ok)
warmup_momentum	0.8	warmup initial momentum
warmup_bias_lr	0.1	warmup initial bias lr
box	0.05	box loss gain
cls	0.3	cls loss gain
cls_pw	1.0	cls BCELoss positive_weight
obj	0.7	obj loss gain (scale with pixels)
obj_pw	1.0	obj BCELoss positive_weight
iou_t	0.20	IoU training threshold
anchor_t	4.0	anchor-multiple threshold
fl_gamma	0.0	focal loss gamma (efficientDet default gamma=1.5)
hsv_h	0.015	image HSV-Hue augmentation (fraction)
hsv_s	0.7	image HSV-Saturation augmentation (fraction)
hsv_v	0.4	image HSV-Value augmentation (fraction)
degrees	0.0	image rotation (+/- deg)
translate	0.1	image translation (+/- fraction)
scale	0.9	image scale (+/- gain)
shear	0.0	image shear (+/- deg)
perspective	0.0	image perspective (+/- fraction), range 0-0.001
flipud	0.0	image flip up-down (probability)
fliplr	0.5	image flip left-right (probability)
mosaic	1.0	image mosaic (probability)
mixup	0.15	image mixup (probability)
copy_paste	0.0	image copy paste (probability)
paste_in	0.15	image copy paste (probability)
